# Interactions of Bovine Serum Albumin with Anti-Cancer Compounds Using a ProteOn XPR36 Array Biosensor and Molecular Docking

**DOI:** 10.3390/molecules21121706

**Published:** 2016-12-10

**Authors:** Ling Zhang, Qiao-Yan Cai, Zhi-Xiong Cai, Yi Fang, Chun-Song Zheng, Li-Li Wang, Shan Lin, Da-Xin Chen, Jun Peng

**Affiliations:** 1 Academy of Integrative Medicine, Fujian University of Traditional Chinese Medicine, Fuzhou 350122, China; remona1986@126.com (L.Z.); cqy2005899@163.com (Q.-Y.C.); icomehere@163.com (Y.F.); zhengcs@foxmail.com (C.-S.Z.); wanglili9268@126.com (L.-L.W.); lisa3350@163.com (S.L.); cdx1125@126.com (D.-X.C.); 2 Fujian Key Laboratory of Integrative Medicine on Geriatrics, Fujian University of Traditional Chinese Medicine, Fuzhou 350122, China; 3 Mengchao Hepatobiliary Hospital of Fujian Medical University, Fuzhou 350025, China; caizhixiong1985@163.com

**Keywords:** surface plasmon resonance, compounds/BSA interaction, kinetics, molecular docking

## Abstract

The aim of the work was to determine the interactions of a set of anti-cancer compounds with bovine serum albumin (BSA) using a ProteOn XPR36 array biosensor and molecular docking studies. The results revealed that a total of six anti-cancer compounds: gallic acid, doxorubicin, acteoside, salvianolic acid B, echinacoside, and vincristine were able to reversibly bind to the immobilized BSA. The sensorgrams of these six compounds were globally fit to a Langmuir 1:1 interaction model for binding kinetics analysis. There were significant differences in their affinity for BSA, with doxorubicin, the weakest binding compound having 1000-fold less affinity than salvianolic acid B, the strongest binding compound. However, compounds with a similar KD often exhibited markedly different kinetics due to the differences in *k_a_* and *k_d_*. Molecular docking experiments demonstrated that acteoside was partially located within sub-domain IIA of BSA, whereas gallic acid bound to BSA deep within its sub-domain IIIA. In addition, the interactions between these compounds and BSA were dominated by hydrophobic forces and hydrogen bonds. Understanding the detailed information of these anti-cancer compounds can provide important insights into optimizing the interactions and activity of potential compounds during drug development.

## 1. Introduction

Numerous small-molecule compounds are capable of binding various target proteins in order to exhibit their antitumor activity. Serum albumin (SA), exemplified by bovine serum albumin (BSA) and human serum albumin (HSA), is the most abundant transport protein in plasma, playing important roles in the absorption, metabolism, pharmacological, and toxicological effects of compounds [1,2]. In order to effectively determine the overall pharmacokinetic profile of small-molecule compounds, it is important to investigate the affinity of compounds on SA. The use of BSA is, thus, considered as a model protein for studying compound-protein interactions due to its low cost, availability, and similarity to HSA [3,4]. Currently, various techniques have been used to study the binding affinity of compounds on proteins in vitro, including equilibrium dialysis, ultrafiltration, spectroscopy, capillary electrophoresis, nuclear magnetic resonance (NMR), surface plasmon resonance (SPR) technology, and molecular docking [5,6,7,8,9,10,11,12,13].

Recently, SPR technology has been developed to detect and analyze changes in the refractive index of buffers, where various molecules interact with the targets immobilized on the surface [14,15]. A ProteOn XPR36 parallel array biosensor based on SPR technology allows for real-time, quantitative detection of biomolecular interactions without the need for labeling and displays increased sensitivity, higher throughput. Therefore, this instrument provides enhanced support for small-molecule compound discovery and development, while being less expensive and time-consuming [16,17,18]. Molecular docking is another important technique which can provide better understanding of the various compound interactions with BSA by identifying the possible binding sites and primary forces involved [11,19,20].

In this study, we utilized the ProteOn XPR36 system to detect the interactions between BSA and a set of anti-cancer compounds including fluorouracil, hydroxytyrosol, gallic acid, matrine, salidroside, curcumin, oxaliplatin, paeoniflorin, doxorubicin, acteoside, ginsenoside Rh1, salvianolic acid B, echinacoside, and vincristine [21,22,23,24,25,26,27,28,29,30]. In addition, we performed molecular docking studies to evaluate the possible binding sites, interaction forces as well as residues of subdomain interaction of acteoside and gallic acid with BSA, respectively. The study can provide important insights into optimizing the interactions and activity of potential compounds during compound development.

## 2. Results

### 2.1. Sensorgrams of Anti-Cancer Compounds

**Figure 1:**
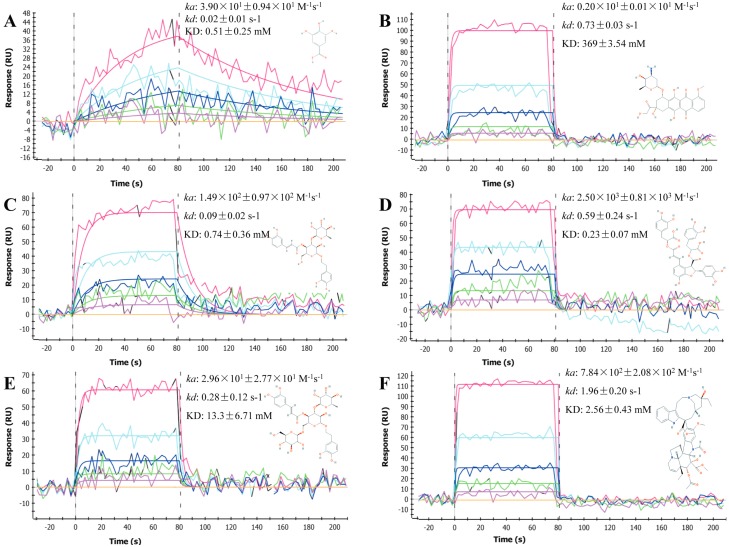
Sensorgrams for the interactions of BSA with the six test compounds; the structure of each compound was shown in the insets. Overlays of the models represented a global fit of the experimental data to a Langmuir 1:1 interaction model. Sensorgrams were representative of three independent experiments. (**A**) gallic acid; (**B**) doxorubicin; (**C**) acteoside; (**D**) salvianolic acid B; (**E**) echinacoside; and (**F**) vincristine.

BSA was immobilized onto the GLH sensor surface via amine coupling, which resulted in final immobilization levels of approximately 11,000 RU (resonance units). Next, the immobilized BSA was interacted with various anti-cancer compounds, with large variations in molecular masses from fluorouracil (130.08 Da) to vincristine (824.96 Da) (Table 1) and different gradient concentrations. All resulting sensorgrams were collected and analyzed using the ProteOn manager software (Figure 1 and Figure S1). We determined that a total of six anti-cancer compounds: gallic acid, doxorubicin, acteoside, salvianolic acid B, echinacoside and vincristine were able to reversibly bind to the immobilized BSA (Figure 1). Interestingly, other than gallic acid, the association phases of these compounds all reached the steady state within a few seconds, while their dissociation phase signals rapidly returned to baseline almost immediately following injection. The sensorgrams of these six compounds were globally fit to a Langmuir 1:1 interaction model (for overlays of modeled and experimental data, see Figure 1), which demonstrated that it was possible to use a simple model to analyze the various number of interactions recorded on the biosensor.

### 2.2. Determination and Comparison of Kinetic Binding Constants

**Figure 2:**
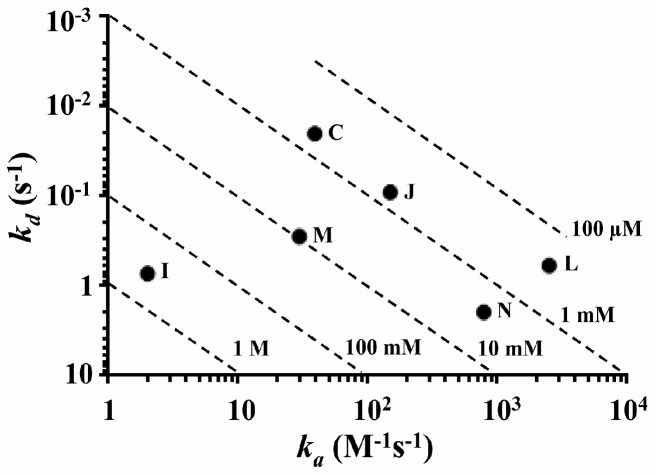
Kinetic profile plot of the six test compounds; Compounds: (**C**) gallic acid; (**I**) doxorubicin; (**J**) acteoside; (**L**) salvianolic acid B; (**M**) echinacoside; and (**N**) vincristine. The diagonal dashed lines represented the equilibrium dissociation constant (KD).

Determination of kinetic binding constants for all six compounds was provided in Figure 1, and the comparisons between their association and dissociation rates were plotted (Figure 2). Association rate was indicated by the *x*-axis, where 1 had the slowest and 10^4^ had the fastest association rate. Dissociation rate was indicated by the *y*-axis, where 10^−3^ had the slowest and 10 had the fastest dissociation rate. Diagonal dashed lines represented the equilibrium dissociation constant (KD), from 1 M to 100 µM. We determined that the difference in affinity between doxorubicin (**I**), the weakest binding compound to salvianolic acid B (**L**), the strongest binding compound was greater than 1000-fold. Vincristine (**N**) had by far the fastest dissociation rate; salvianolic acid B (**L**), doxorubicin (I) and echinacoside (**M**) had intermediate dissociation rates, whereas acteoside (**J**) and gallic acid (**C**) had the slowest dissociation rates. Compounds with similar KD often displayed observable differences in their kinetics, as indicated by their *k_a_* and *k_d_*. For instance, acteoside (**J**) and gallic acid (**C**) bound to BSA with approximately the same KD; however, these two compounds exhibited markedly different kinetics, where acteoside had significantly faster *k_a_* and *k_d_* compared to gallic acid.

### 2.3. Molecular Docking Studies

We demonstrated above that six anti-cancer compounds were able to reversibly bind to the immobilized BSA. In order to evaluate the possibility that additional compounds could reversibly bind to BSA, molecular docking was carried out using LibDock protocol, with Discovery Studio 2.5 software. We selected acteoside and gallic acid as reference compounds in order to identify the possible binding sites on BSA, and to better understand the interaction mechanisms. And the results of other compounds were provided in Figures S2–S5. BSA is a globular protein which contains 583 amino acids and consists of three homologous domains (I, II, and III): I (residues 1–183), II (184–376), III (377–583), each containing two sub-domains (A and B). Two hydrophobic cavities are present in sub-domain IIA and IIIA, which form the site of binding for various compounds [31,32].

**Figure 3:**
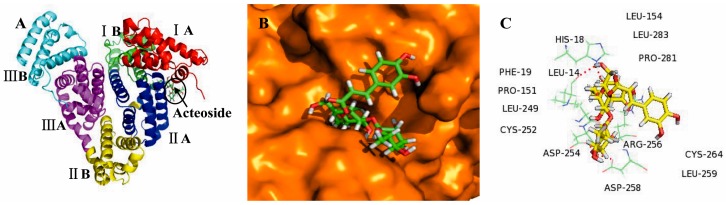
(**A**) The docking conformation of acteoside-BSA complex with the lowest energy conformation; the BSA and acteoside were represented in the cartoon as indicated; (**B**) Molecular docking model of acteoside partially located within sub-domain IIA of BSA; BSA and acteoside were represented by the orange sphere model and green stick model, respectively; (**C**) The surrounding hydrophobic amino acid residues within 6 Å and hydrogen bond interactions between acteoside and BSA; hydrogen bonds, amino acids, and acteoside were represented by red dashed lines, green lines, and yellow stick model, respectively.

**Figure 4:**
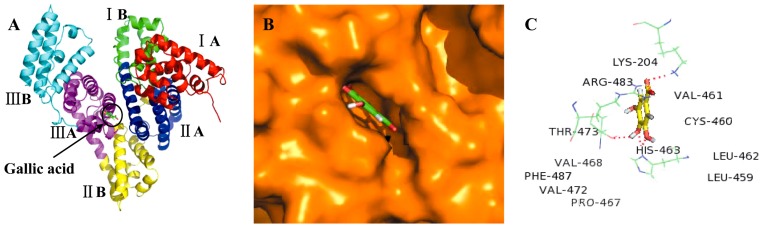
(**A**) The docking conformation of gallic acid-BSA complex with the lowest energy conformation; the BSA and gallic acid were represented in the cartoon as indicated; (**B**) Molecular docking model of gallic acid deeply inserted into the sub-domain IIIA of BSA; BSA and gallic acid were represented by the orange sphere model and green stick model, respectively; (**C**) The surrounding hydrophobic amino acid residues within 6 Å and hydrogen bond interactions between gallic acid and BSA; hydrogen bonds, amino acids and gallic acid were represented by red dashed lines, green lines, and yellow stick model, respectively.

We showed that the best energy conformation model of acteoside was partially located within sub-domain IIA of BSA (Figure 3A,B). Notably, acteoside was surrounded by various hydrophobic residues including Leu-154, Leu-249, Leu-259, Leu-283, Pro-281, Pro-151, Cys-252, Cys-264, and Phe-19. The molecular docking results also indicated that there were hydrogen bonding interactions of acteoside with Arg-256, Asp-254, Asp-258, Leu-14, and His-18 residues (Figure 3C). In contrast, gallic acid bound deep within the pocket at sub-domain IIIA of BSA, in the minimum energy conformation (Figure 4A,B). Gallic acid was also adjacent to many hydrophobic residues including Val-461, Val-468, Val-472, Leu-459, Leu-462, Cys-460, Phe-487, and Pro-467. In addition, there were four hydrogen bonding interactions of gallic acid with Lys-204, His-463, Thr-473, and Arg-483 (Figure 4C). Therefore, we can conclude that acteoside and gallic acid could both bind to BSA, and the interactions between them were dominated by hydrophobic forces and hydrogen bonds. These molecular docking results were consistent with data obtained from SPR technology and, therefore, can provide a solid structural basis for understanding ligand–protein binding interactions.

## 3. Discussion

SPR technology is a useful analytical technology for detecting interactions between various biomolecules, via relaying changes in the refractive index at the surface of a gold chip. Recently, SPR technology has been used commercially through advanced systems such as Biacore S51 and ProteOn XPR36, which are capable of detecting minute changes in the mass of analytes binding to the chip at a temporal resolution of only 0.1 s [33,34]. In this study, we used the ProteOn XPR36 system to determine the interactions of a set of anti-cancer compounds with BSA. It is commonly accepted that the value of KD is positively correlated to temperature [16]. Therefore, variations in temperature which can result in deviations of KD should be avoided via simultaneous injection of analytes at various concentrations. In this regard, the ProteOn XPR36 system can accurately measure concentration-dependent binding data of six different concentrations of analytes simultaneously flowing over the immobilized targets. Perhaps the most important advantage was that this system eliminated the need to regenerate the surface, and allowed the simple fit of all test compounds with the reaction surfaces into a 1:1 interaction model. A possible limitation was that it was difficult to determine the KD of compounds with multiple binding sites.

Generally, the weak interactions of compounds with serum albumin lead to a short lifetime or the poor distribution of compounds. In contrast, the strong binding displays the reverse effect [20]. The weak/strong affinity of compound-BSA is preliminarily evaluated by KD that lower of KD value indicates higher affinity. However, compounds with a similar KD often exhibit markedly different kinetics due to the differences in *k_a_* and *k_d_*, which are also key factors from a pharmacological point of view. As the potential anti-cancer agent, the kinetic binding constants of compounds should be comprehensively considered. Therefore, the selection of anti-cancer compounds is depended on not only the relatively low of KD value, but also weak *k_d_* and strong *k_a_*, which acteoside and gallic acid exhibited this feature among these compounds.

However, there were a few inconsistencies between previous research and our results on the interactions of BSA with the tested anti-cancer compounds [12,35,36,37,38]. In general, they use UV-VIS spectroscopy, fluorescence spectroscopy, Fourier transform infrared (FTIR), circular dichrosim (CD), and mass spectrometry to detect the interactions of compounds with BSA. The principle of each methodology is different. The determination of interactions between compounds with BSA relies on changes in the mass-to-charge ratio by mass spectrometry. Meanwhile, variations in light or band absorption, as well as shifting of the maximum peak position or maximum emission wavelength of compound-BSA were used to reflect the interactions of compounds with BSA via spectroscopic methodologies. For instance, Cheng reported that salidroside could bind to BSA via spectroscopic investigation, whereas our study identified no such binding using SPR technology [12]. Cheng had studied the interaction of salidroside with BSA under physiological conditions, whereas in our study the determination of BSA binding affinity was carried out in PBS-T buffer (10 mM Na-phosphate, 150 mM NaCl, and 0.005% Tween 20, pH 7.4). Therefore, a possible reason was the use of different methodologies and experimental conditions which resulted in the inconsistency. Further studies carried out under various conditions are required to confirm the actual binding affinity of salidroside to BSA.

Molecular docking studies were performed to determine the possible binding sites between the test compounds with BSA, and to provide a molecular explanation for these binding interactions. Using molecular modeling, we determined that the interactions between the tested anti-cancer compounds and BSA were dominated by hydrophobic forces and hydrogen bonds. In addition, we found that the test compounds bound to sub-domain IIA and sub-domain IIIA of BSA, and these sites have been recognized to play important roles in absorption, metabolism and transportation of various compounds. Taken together, studying the interactions between anti-cancer compounds with BSA via SPR technology and molecular docking techniques could provide significant contributions to the improved understanding and future pharmaceutical applications of these compounds.

## 4. Materials and Methods

### 4.1. Materials

All experiments were performed using ProteOn XPR36 instruments from Bio-Rad. A GLH Sensor chip, 50% glycerol, sodium acetate, *N*-ethyl-*N*′-(3-dimethylaminopropyl)carbodiimide (EDC), sulfo-*N*-hydroxysuccinimide (sulfo-NHS) and ethanolamine-HCl were purchased from Bio-Rad (Hercules, CA, USA). Buffer reagents and BSA were purchased from Sigma-Aldrich (St. Louis, MO, USA). Fluorouracil, hydroxytyrosol, gallic acid, matrine, salidroside, curcumin, oxaliplatin, paeoniflorin, doxorubicin, acteoside, ginsenoside Rh1, salvianolic acid B, echinacoside, and vincristine were all purchased from the National Institutes for Food and Compound Control (Beijing, China).

### 4.2. Initialization of GLH Sensor Chip

The GLH sensor chip, consisting of glass prisms coated with gold and an alginate-based layer, was an ideal choice for examining protein-small molecule interactions. The chip was initialized with 50% glycerol for several minutes prior to use.

### 4.3. Preconditioning of GLH Sensor Chip

Following initialization, the GLH sensor chip was washed with 0.5% SDS, 50 mM NaOH and 100 mM HCl, respectively, for 1 min each, at a flow rate of 100 µL/min. PBS-T buffer (10 mM sodium phosphate, 150 mM NaCl, and 0.005% Tween 20, pH 7.4) was then injected in three pulses for baseline stabilization.

### 4.4. Immobilization of BSA

One of the six channels was activated with a mixture of EDC (40 mM) and sulfo-NHS (10 mM) in the horizontal direction for 3 min, at a flow rate of 30 µL/min. BSA (2 mg/mL) was diluted to 300 nM in 10 mM sodium acetate (pH 4.0) and immobilized on the channel for 5 min, at a flow rate of 30 µL/min. Next, the channel was blocked by injection of 1 M ethanolamine-HCl for 5 min. The final immobilization level was approximately 11,000 RU. Baseline equilibration was performed by multiple washes with running buffer (PBS-T) across the chip surface for 1 h. 

### 4.5. Injection of Analytes

The highest concentrations of fluorouracil, hydroxytyrosol, gallic acid, matrine, salidroside, curcumin, oxaliplatin, paeoniflorin, doxorubicin, acteoside, ginsenoside Rh1, salvianolic acid B, echinacoside, and vincristine were 1920, 1500, 500, 200, 1000, 200, 251.7, 200, 36.8, 200, 100, 200, 800, and 400 µM, respectively. Each compound was tested at six concentrations using a two-fold dilution series. Gradient concentrations of each compound were simultaneously injected over the BSA-immobilized surface at a flow rate of 30 µL/min in the vertical direction, where the association and dissociation times were 80 s and 60 s, respectively. All interaction analyses were performed at 25 °C.

### 4.6. Data Processing and Analysis

All sensorgrams for protein binding were collected, processed, and analyzed using the ProteOn Manager software. We utilized two types of reference sources, the standard blank buffer reference, and interspot reference, which was the average response before and after each interaction spot to correct for nonspecific binding. The target protein was washed off in the dissociation phase, which caused baseline drift, although real-time injection of a blank reference can completely correct this baseline drift by subtracting artifacts in real time. The dual references were both subtracted from the interaction spot data to correct for any bulk shifts due to baseline drift and nonspecific binding. Finally, the data for each compound collected over the same target protein were globally fit to a Langmuir 1:1 binding model with a global *k_a_* and *k_d_*, where *k_a_* is the association rate constant and *k_d_* is the dissociation rate constant for the protein-small molecule binding. The ratio of (*k_d_*/*k_a_*) indicated the value for the equilibrium dissociation constant (KD).

### 4.7. Molecular Docking

The structures of acteoside and gallic acid were obtained from the PubChem Compound Database for the molecular docking study. The MMFF force field was utilized for the energy minimization of compounds using Discovery Studio 2.5 software. The crystal structure of BSA (PDB ID: 4F5S) was obtained from RCSB Protein Data Bank. Binding interactions of test compounds with BSA were simulated by molecular docking using LibDock protocol with Discovery Studio 2.5 software. BSA was prepared by the removal of all water molecules and heteroatoms, and addition of hydrogen atoms. According to the scoring function, the compound–BSA complex conformation with the lowest binding energy was used for subsequent analysis. Molecular docking results were illustrated using the PyMol molecular graphic program.

## Figures and Tables

**Table 1:** Compounds used in analysis.

Sample	Compound	Molecular Mass (Da)
A	**Fluorouracil**	130.08
B	**Hydroxytyrosol**	138.16
C	**Gallic acid**	170.12
D	**Matrine**	248.37
E	**Salidroside**	300.31
F	**Curcumin**	368.39
G	**Oxaliplatin**	397.29
H	**Paeoniflorin**	480.45
I	**Doxorubicin**	543.52
J	**Acteoside**	624.59
K	**Ginsenoside Rh1**	638.87
L	**Salvianolic acid B**	718.62
M	**Echinacoside**	786.72
N	**Vincristine**	824.96

## Supplementary Materials

The following are available online at http://www.mdpi.com/1420-3049/21/12/1706/s1.

## Abbreviations

SPR	surface plasmon resonance
*k_a_*	association rate constant
*k_d_*	dissociation rate constant
KD	equilibrium dissociation constant
SA	Serum albumin
HSA	human serum albumin
BSA	bovine serum albumin
EDC	*N*-ethyl-*N*′-(3-dimethylaminopropyl)carbodiimide
sulfo-NHS	sulfo-*N*-hydroxysuccinimide
RU	Resonance Units
