# Supplementary material for: Interactions of Bovine Serum Albumin with Anti-Cancer Compounds Using a ProteOn XPR36 Array Biosensor and Molecular Docking
Source: Molecules. 2016 Dec 10;21(12):1706. doi: 10.3390/molecules21121706 (PMC6274227; doi:10.3390/molecules21121706)
Supplement: Supplementary file 1 [file molecules-21-01706-s001.pdf]

Supplementary Materials: Interactions of Bovine Serum Albumin with Anti-Cancer Compounds Using a ProteOn XPR36 Array Biosensor and Molecular Docking

Ling Zhang, Qiao-Yan Cai, Zhi-Xiong Cai, Yi Fang, Chun-Song Zheng, Li-Li Wang, Shan Lin, Da-Xin Chen and Jun Peng

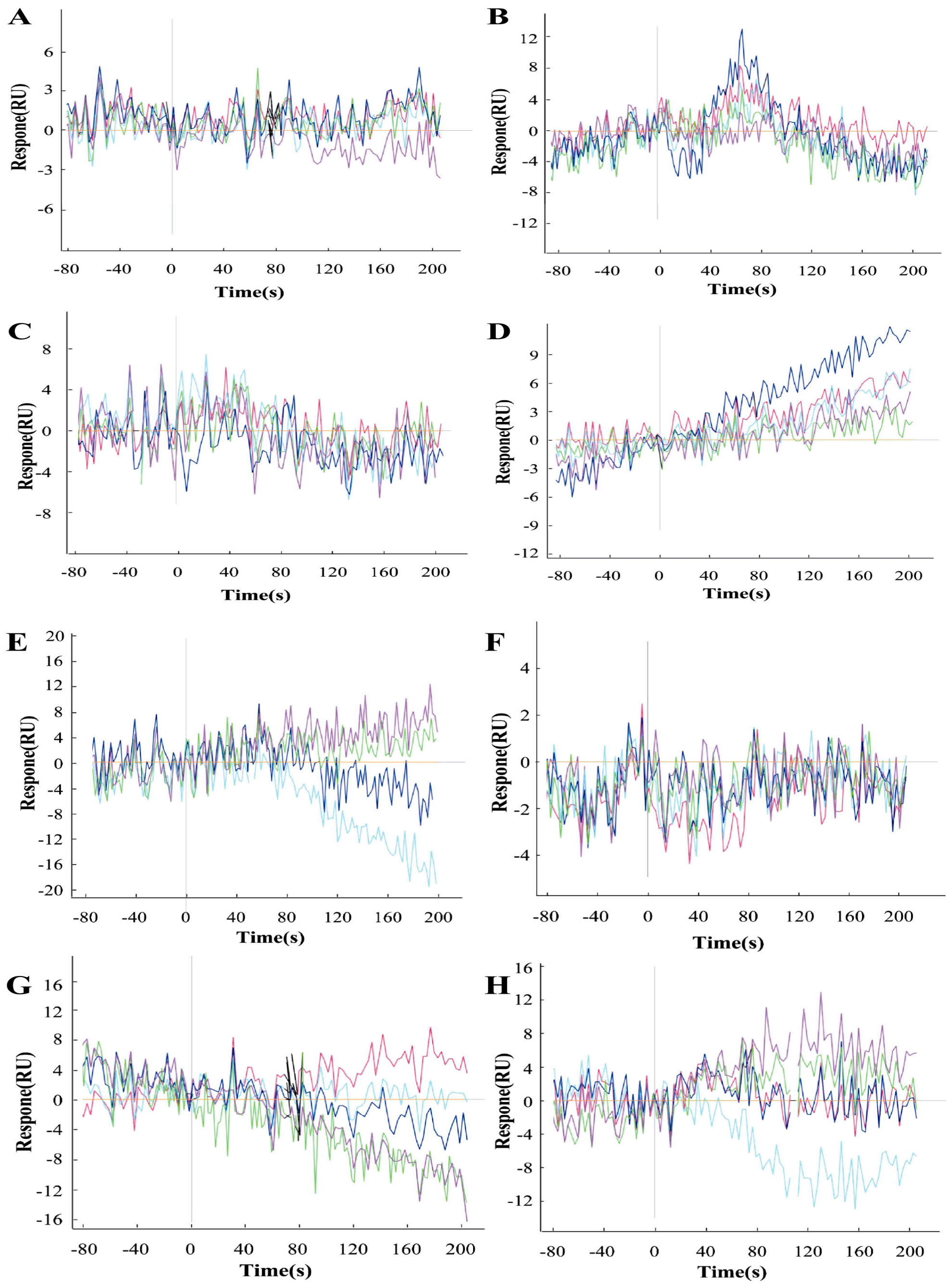

Figure S1. Sensorgrams for the interactions of bovine serum albumin (BSA) with the eight test compounds; (**A**) fluorouracil; (**B**) hydroxytyrosol; (**C**) matrine; (**D**) salidroside; (**E**) curcumin; (**F**) oxaliplatin; (**G**) paeoniflorin; (**H**) ginsenoside Rh1.

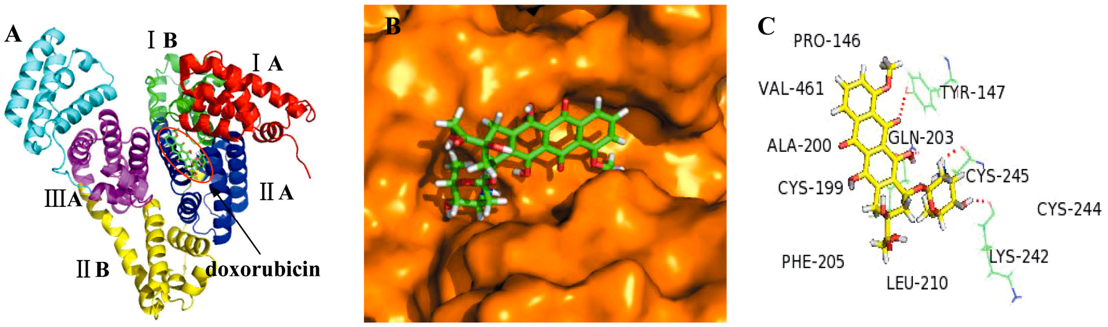

Figure S2. (**A**) The docking conformation of doxorubicin-BSA complex with the lowest energy conformation; The BSA and doxorubicin were represented in the cartoon as indicated; (**B**) Molecular docking model of doxorubicin partially located within sub-domain IIA of BSA; BSA and doxorubicin were represented by the orange sphere model and green stick model, respectively; (**C**) The surrounding hydrophobic amino acid residues within 6 Å and hydrogen bond interactions between doxorubicin BSA; hydrogen bonds, amino acids, and doxorubicin were represented by red dashed lines, green lines, and yellow stick model, respectively.

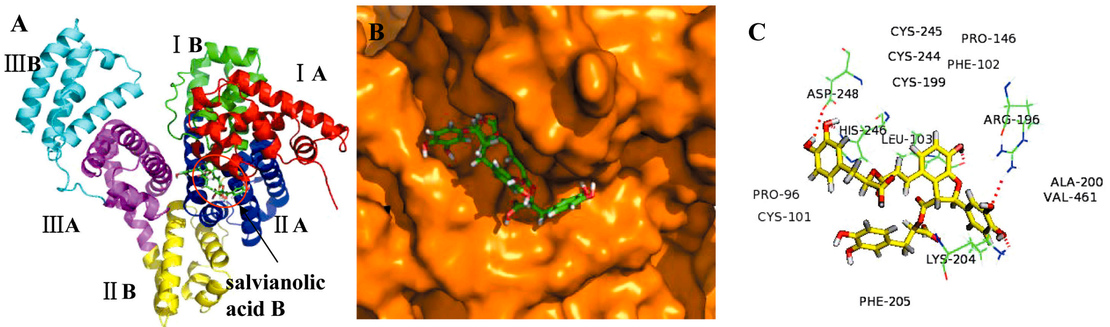

Figure S3. (**A**) The docking conformation of salvianolic acid B-BSA complex with the lowest energy conformation; the BSA and salvianolic acid B were represented in the cartoon as indicated; (**B**) Molecular docking model of salvianolic acid B inserted into the sub-domain IIA of BSA; BSA and salvianolic acid B were represented by the orange sphere model and green stick model, respectively; (**C**) The surrounding hydrophobic amino acid residues within 6 Å and hydrogen bond interactions between salvianolic acid B BSA; hydrogen bonds, amino acids, and salvianolic acid B were represented by red dashed lines, green lines, and yellow stick model, respectively.

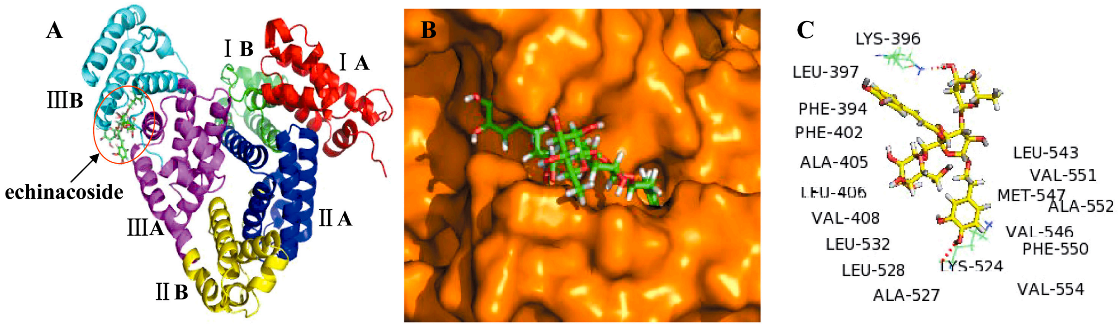

Figure S4. (**A**) The docking conformation of echinacoside-BSA complex with the lowest energy conformation; the BSA and echinacoside were represented in the cartoon as indicated; (**B**) Molecular docking model of echinacoside partially located within sub-domain IIIA of BSA; BSA and echinacoside were represented by the orange sphere model and green stick model, respectively; (**C**) The surrounding hydrophobic amino acid residues within 6 Å and hydrogen bond interactions between echinacoside BSA; hydrogen bonds, amino acids, and echinacoside were represented by red dashed lines, green lines, and yellow stick model, respectively.

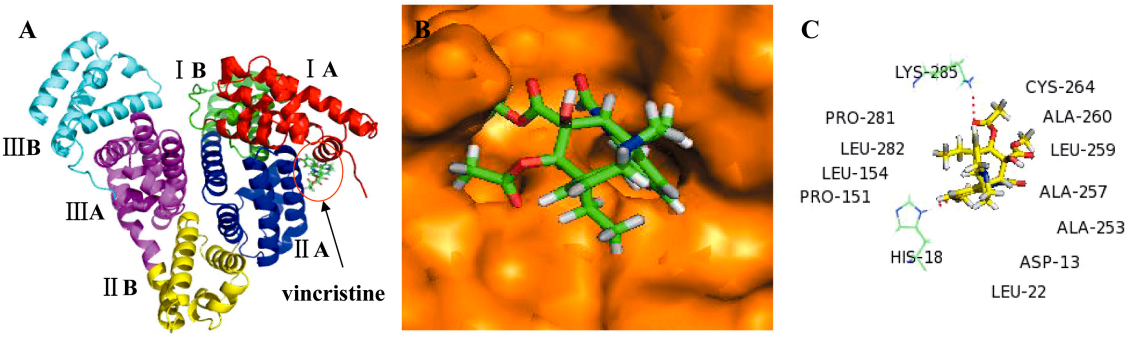

Figure S5. (**A**) The docking conformation of vincristine-BSA complex with the lowest energy conformation; the BSA and vincristine were represented in the cartoon as indicated; (**B**) Molecular docking model of vincristine partially located within sub-domain IIA of BSA; BSA and vincristine were represented by the orange sphere model and green stick model, respectively; (**C**) The surrounding hydrophobic amino acid residues within 6 Å and hydrogen bond interactions between vincristine BSA; hydrogen bonds, amino acids, and vincristine were represented by red dashed lines, green lines, and yellow stick model, respectively.
